# Discovery of a Symptomatic Left Anomalous Coronary Artery from the Opposite Sinus and Postoperative Considerations

**DOI:** 10.1155/2009/509064

**Published:** 2009-10-15

**Authors:** Ahmad Slim, John Thurlow, Jennifer Blevins, Shaun Martinho, Brian Markelz

**Affiliations:** Brooke Army Medical Center, San Antonio, TX 78234, USA

## Abstract

This is the case of an 18 year old active duty soldier with symptoms of exertional chest pressure and syncope who was found to have anomalous origin of the left main coronary artery (LMCA) from the right coronary cusp (RCC) traveling partially between the great vessels before taking a septal approach between the left ventricular outflow tract (LVOT) and the right ventricular outflow tract (RVOT). Anomalous origin of coronary arteries is a rare condition that carries an increased risk of angina, myocardial ischemia, and sudden cardiac death (SCD). Surgical treatment of such anomalies with both high and lower risk features can be challenging, and traditional benefit from surgical correction may not be achieved due to complex anatomy. As evident by our patient, this rare condition even though benign from sudden death standpoint could be debilitating despite best efforts and available resources.

## 1. Case Report

This is the case of an 18-year-old active duty soldier with symptoms of severe gastroenteritis and exertional chest pressure that radiated to his neck and left arm relieved by rest. These symptoms prevented him from participating in sports throughout high school. Although, the patient described one episode of near syncope following a two-mile run in which he became very lightheaded but never lost consciousness. 

**Figure 1:**
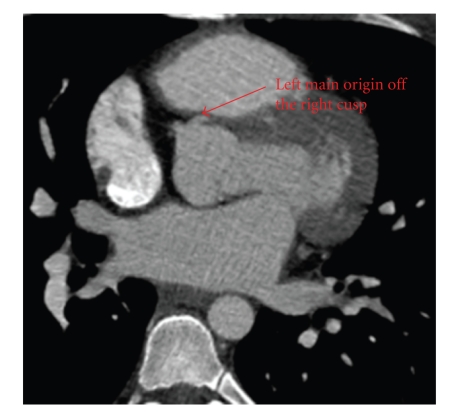
Coronary CT showing the origin of the left main (LM) from the right coronary cusp.

**Figure 2:**
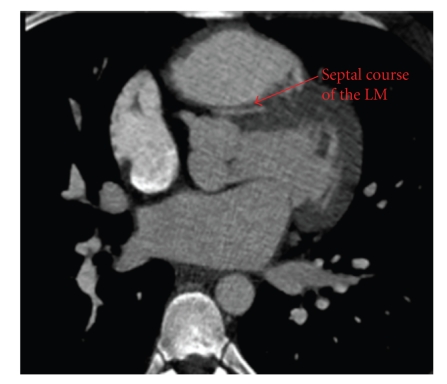
Coronary CT showing the left main (LM) traveling between the left ventricular outflow tract (LVOT) and the right ventricular outflow tract (RVOT).

**Figure 3:**
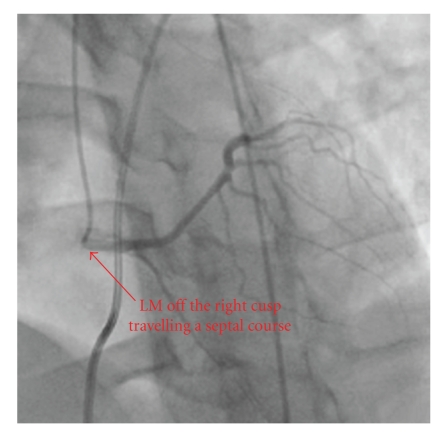
Coronary angiography showing the left main (LM) originating from the right cusp and traveling between the left ventricular outflow tract (LVOT) and the right ventricular outflow tract (RVOT).

 Imaging evaluation began with a transthoracic echocardiogram which revealed a normal left ventricular ejection fraction with no regional wall motion abnormality. With the patient history and presentation, a subsequent ECG-gated cardiac CT angiogram was obtained to evaluate the coronary artery origins. This study demonstrated an anomalous origin of the left main coronary artery (LMCA) from the right coronary cusp (RCC) travelling partially between the great vessels before taking a septal approach between the left ventricular outflow tract (LVOT) and the right ventricular outflow tract (RVOT) (see Figures 1 and 2). Coronary angiography ruled out additional obstruction and confirmed the anomalous course of the LMCA between the aorta and RVOT followed by a septal course (see Figure 3). On the basis of his cardiac symptoms and anomalous coronary artery anatomy, he was directly referred for immediate evaluation by a specialized congenital center as part of multicenter collaboration. Patient's anatomy limited the possibility of reanastomosis or pulmonary patch, leaving the surgeon no option but single vessel bypass to left anterior descending (LAD) by the left internal mammary artery (LIMA). On follow up, the patient continued to have exertional symptoms and treadmill myocardial perfusion scan where patient exercised for 12 minutes with chest pain at peak exercise showed lateral wall ischemia. An elective coronary angiography showed patent LIMA to left anterior descending artery with normal FFR. Most likely, patient's symptoms are related to the unrevascularized territory of the left circumflex system. 

## 2. Discussion

The overall incidence of reported anomalous coronary arteries during coronary angiography is 5.64% Specifically, the incidence of the LMCA originating off of the right coronary cusp (RCC) is 0.15% [1]. Sudden cardiac death among young athletes has a prevalence of 0.5 : 100 000 in the US and 20% of these deaths are attributed to congenital coronary artery anomalies. Patients with anomalous aortic origin of a coronary artery comprise the majority of mortality in this high-risk group [2]. Anomalous LMCA origins tend to have more significant symptoms, while those with anomalous RCA origin tend to be silent. Symptoms due to these anomalies are presumed to be due to a variety of causes. Often, the ostia of these anomalous arteries have a slit-like orifice, and the abnormal angles of these arteries could lead to kinking. Both of these findings would limit blood flow and induce ischemia. Arrhythmias could be caused by myocardial scaring from this chronic ischemic damage [3].

The anatomic course of an anomalous LMCA beyond the origin is what determines the extent of symptoms and risk. Traditionally, when an LMCA originates from the RCC, it takes one of four courses: retroaortic, anterior free wall, interarterial, or septal [4]. Eighty percent of these patients with sudden cardiac death are found to have an intra-arterial pathway, while the other pathways are considered more benign [2]. However, case reports documenting unstable angina, ventricular tachycardia, and syncope in patients with septal courses are challenging the benign labeling of septal course LMCA [4]. 

Identification of anomalous origin of coronary arteries is highly related to the index of clinical suspicion. We can presume that current suspicion is generally low, given that sudden cardiac death is often the symptom that alludes to the diagnosis [3]. In a recent small retrospective study, clinical symptoms were present in 45% of patients prior to their sudden cardiac death [3]. Per current AHA/ACC guidelines, patients with unexplained aborted sudden cardiac death, unexplained life-threatening arrhythmia, unexplained coronary ischemic symptoms, or unexplained LV dysfunction have a class I indication to screen for anomalous coronary arteries. The current screening exam of choice would be CT or MR angiography [2].

Once identified, the patient should be placed on strict bed rest, and prompt surgical evaluation, by centers with experience in treating anomalous coronary arteries, should occur [3, 5]. Surgical treatment is the first line for patients at high risk for SCD; although, surgery may also be offered to lower risk asymptomatic patients as well. Treating both symptomatic and asymptomatic anomalies have not shown to negatively impact the quality of life [6]. A recent small study challenges the security of surgical treatment by demonstrating subclinical ischemic findings which are common after surgical repair of anomalous aortic coronary origin, even in patients with patent neocoronary ostia [5].

The typical surgical modality for an interarterial course involves relocation of the anomalous artery from between the great vessels to around them in order to remove the compressive shear forces of the great vessels on the coronary artery. More conservative medical management is used for patients with a septal course. Our patient's anomaly followed a septal course where conservative approach would have been recommended but due to severity of symptoms surgical intervention was deemed prudent. Relocation of the artery would have been the ideal surgical procedure, but with most of the LM course septaly, the only available option was bypass grafting with LIMA to LAD. Patient's left circumflex anatomy with no obtuse marginal targets limited bypass grafting to this territory. Given competitive flow, graft patency and maturation, even in the setting of an arterial graft, was of great concern. However, arterial graft matured as was evident on repeat angiography and patient's persistent symptoms were most likely related to the lateral wall unrevascularized territory. Patient's septal course is deemed benign from sudden cardiac death perspective and thus no intracardiac defibrillator placement was attempted.

## 3. Conclusion

Anomalous origin of coronary arteries is a rare condition that carries an increased risk of angina, myocardial ischemia, and sudden cardiac death (SCD). This condition does not have a high prevalence in the US, however, when present can have catastrophic results [4]. Many patients are identified on postmortem exam, indicating that a higher index of suspicion along with complete imaging is needed to identify and treat patients before complications arise [3]. Current treatment involves strict cessation of exercise activity until surgical intervention is undertaken if warranted [2].

Surgical treatment of such anomalies with both high and lower risk features can be challenging, and traditional benefit from surgical correction may not be achieved due to complex anatomy. As evident by our patient, this rare condition even though benign from sudden death standpoint could be debilitating despite best efforts and available resources.

## References

[1] Angelini P (1999). *Coronary Artery Anomalies: A Comprehensive Approach*.

[2] Warnes CA, Williams RG, Bashore TM (2008). ACC/AHA 2008 guidelines for the management of adults with congenital heart disease. *Circulation*.

[3] Basso C, Maron BJ, Corrado D (2000). Clinical profile of congenital coronary artery anomalies with origin from the wrong aortic sinus leading to sudden death in young competitive athletes. *Journal of the American College of Cardiology*.

[4] Barry MO, Seeck BA, Virgilio C (2008). Left main coronary anomaly arising from the right sinus of valsalva-interarterial, septal, or a continuum?. *Journal of Thoracic Imaging*.

[5] Brothers JA, McBride MG, Seliem MA (2007). Evaluation of myocardial ischemia after surgical repair of anomalous aortic origin of a coronary artery in a series of pediatric patients. *Journal of the American College of Cardiology*.

[6] Brothers JA, McBride MG, Marino BS (2009). Exercise performance and quality of life following surgical repair of anomalous aortic origin of a coronary artery in the pediatric population. *The Journal of Thoracic and Cardiovascular Surgery*.

